# Complete mitochondrial genome of *Dioryctria yiai* (Lepidoptera: Pyralidae)

**DOI:** 10.1080/23802359.2020.1721352

**Published:** 2020-02-06

**Authors:** Yu-Peng Wu, Jun-Jiao Lu, Jing Yang, Ren-Jun Fan

**Affiliations:** a College of Environment and Safety, Taiyuan University of Science and Technology, Taiyuan, China;; b Shanxi Key Laboratory of Integrated Pest Management in Agriculture, Institute of Plant Protection, Shanxi Academy of Agricultural Sciences, Taiyuan, China

**Keywords:** *Dioryctria yiai*, complete mitogenome, Pyralidae

## Abstract

The *Dioryctria yiai* belongs to Pyralidae in Lepidoptera. The complete mitogenome of *D. yiai* was described in this study, which is typical circular duplex molecules and 15,430 bp in length, containing the standard metazoan set of 13 protein-coding genes, 22 transfer RNA genes, 2 ribosomal RNA genes, and an A + T-rich region. The gene order is same with other lepidopterans. Except for *cox1* started with CGA, all other PCGs started with the standard ATN codons. Most of the PCGs terminated with the stop codon TAA, whereas *cox2* has the incomplete stop codon T. The phylogenetic tree showed that *D. yiai* and other six species belong to Phycitinae, are clustered into a clade.

*Dioryctria yiai* is a species of snout moth in the genus Dioryctria and is known from Taiwan and China. The larvae feed on *Pinus massoniana* and damage the branches, cones and shoots of host plant. The species overwinters in the larval stage within the damaged branch, cone or shoot. At present, its mitochondrial genome has not been reported publicly.

In this study, the samples were collected by light trapping in Taiyuan city of China (37°83′33″N, 112°66′61″E) in July 2019, some of these specimens were immediately frozen at −80 °C on board for mitogenome analysis and others were preserved by spreading wings in the Herbarium of Institute of Plant Protection, Shanxi Academy of Agricultural Sciences and their numbers is 2019TYKD1711-1715. Total genomic DNA was extracted from tail tip using the Ezup pillar genomic DNA extraction kit (Sangon Biotech, Shanghai, China). The mitogenome was sequenced by Illumina Hiseq 4000. Gene annotation was performed and circularity was checked using the MITOS2 webserver (Bernt et al. 2013, http://mitos.bioinf.uni-leipzig.de/).

The mitochondrial genome of *D. yiai* has a total length of 15,430 bp (GenBank accession No. MN658208), consisting of 13 PCGs, 22 tRNA, 2 rRNA genes, and an A + T-rich region. The major strand encodes a larger number of genes (9 PCGs and 14 tRNAs) than the minor strand (4 PCGs, 8 tRNAs, and 2 rRNA genes). Gene content and arrangement are highly conserved and typical of Lepidoptera (Wu et al. 2016). The mitogenome is highly biased toward A/T, contains 42.02% T, 38.99% A, 13.34% C, and 7.65% G, which is a feature commonly present in insects (Boore 1999).

All of the protein**-**coding genes have ATN as the start codon except for *cox1*, which starts with CGA. Eleven PCGs have the common stop codon TAA, *cox2* has the incomplete stop codon T. All tRNAs exhibit typical clover-leaf secondary structure, except for tRNA-Ser(AGN) lacking the DHU arm, which is common in Lepidoptera insects (Garey and Wolstenholme 1989). The 16S rRNA is 1440 bp in length and the 12S rRNA is 795 bp in length. The A + T-rich region is 329 bp long located between 12S rRNA and tRNA-Met and it is longer than other most Lepidoptera insects. There is a motif ATAGA in downstream of 12S rRNA followed by an 18 bp Poly-T stretch.

The phylogenetic position of *D. yiai* was inferred using sequences of the 13 PCGs of 12 species. Eleven of them belong to Pyralidae and a species from Noctuidae (which is used as outgroup) (Figure 1). The sequences were aligned with MAFFT v7.2 software (Katoh and Standley 2013), the evolutionary analyses were conducted with RAxML v8.2.10 (Stamatakis 2014) on the CIPRES Science Gateway (Miller et al. 2010). The result showed that *D. yiai* and other six species belong to Phycitinae, are clustered into a clade. Galleriinae include two species *Corcyra cephalonica* and *Galleria mellonella* and form a monophyly. Epipaschiinae (represented by *Lista haraldusalis*) is close to Pyralinae ((represented by *Hypsopygia regina*).

**Figure 1.**
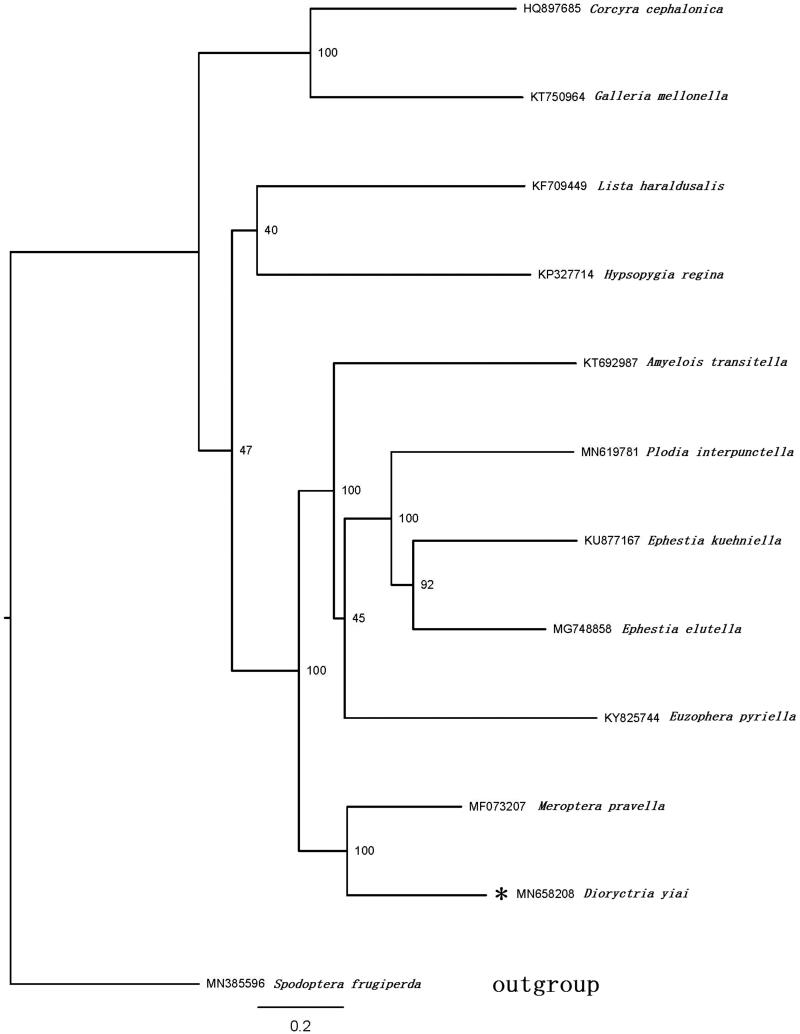
Maximum-likelihood tree of evolutionary relationships *D. yiai* based on the complete mitogenomes of 12 Lepidopteran moths. The marked * is the sample sequence in this study.

## Nucleotide sequence accession number

The complete mitochondrial genome sequence of *D. yiai* was deposited in GenBank under the accession number MN658208.
